# Complete Genome Sequence of *Legionella sainthelensi* Isolated from a Patient with Legionnaires’ Disease

**DOI:** 10.1128/genomeA.01588-17

**Published:** 2018-02-01

**Authors:** Sandy Slow, Trevor Anderson, Patrick Biggs, Martin Kennedy, David Murdoch, Simone Cree

**Affiliations:** a Department of Pathology and Biomedical Science, University of Otago, Christchurch, New Zealand; b Microbiology, Canterbury Health Laboratories, Christchurch, New Zealand; c Molecular Epidemiology and Veterinary Public Health Laboratory, Infectious Disease Research Centre, Institute of Veterinary, Animal and Biomedical Science, Massey University, Palmerston North, New Zealand

## Abstract

*Legionella sainthelensi* is an aquatic environmental bacterium that in humans can cause Legionnaires’ disease (LD), an often severe form of pneumonia. Here, we report the first complete genome of a *L. sainthelensi* clinical isolate obtained in 2001 from a patient with LD in Canterbury, New Zealand.

## GENOME ANNOUNCEMENT

*Legionella* spp. are ubiquitous Gram-negative intracellular bacterial pathogens that predominantly inhabit aquatic environments. Human infections by *Legionella* bacteria manifest clinically either as a self-limiting, flu-like illness called Pontiac fever or as an often severe form of pneumonia known as Legionnaires’ disease (LD). Of the more than 60 different species in the *Legionella* genus, nearly half have been implicated in human disease (1, 2). While the most clinically relevant species are *L. pneumophila* and *L. longbeachae* (3, 4), improvements in diagnostic testing using molecular tools have found that a small minority of cases are caused by other species such as *L. sainthelensi*. In 1981, *L. sainthelensi* was first documented in cultures of freshwater sampled near the Mt. St. Helens volcano (5); however, it was not until 1990 that it was isolated from patient respiratory specimens (6), providing evidence that it could cause human disease. Currently, there are two draft genome sequences for the ATCC 35248 type strain, which are the environmental isolates obtained near the Mt. St. Helens volcano (GenBank accession numbers NZ_JHXP00000000 and NZ_LNYV00000000). Here, we report the first complete genome sequence of a *L. sainthelensi* clinical isolate obtained from a patient with LD in 2001 from Christchurch, New Zealand.

The isolate was grown on buffered charcoal-yeast extract agar (72 h, 35°C), and DNA was purified using the Genomic-Tip 100/G kit (Qiagen, Hilden, Germany). Sequencing was conducted using the MinION Nanopore (Oxford Nanopore Technologies, United Kingdom) and Illumina MiSeq (San Diego, CA, USA) systems. For MinION library preparation, 5 µg of genomic DNA, quantified by Qubit fluorometry (Thermo, Fisher Scientific, Waltham, MA, USA), underwent end repair and deoxyadenosine monophosphate (dA) tailing using the NEBNext Ultram end repair/dA tailing module (New England Biolabs, Ipswich, MA, USA), followed by adaptor ligation and purification of double-stranded DNA with hairpin adaptors using the Nanopore MinION genomic sequencing kit (R9 flow-cell chemistry). The library was sequenced using MinKNOW software for 48 h. Additional sample mix was applied to the flow cell when the number of pores being used was less than 20, until the entire sample was used. The raw electrical signal was uploaded to Metrichor (version 1.107), using the two-directional base-calling recurrent neural network for SQK-NSK007. The MiSeq library (250-bp paired-end reads) was prepared using the Nextera XT protocol and sequenced using V2 chemistry. Approximately 30,000 two-directional high-quality MinION reads and 1,600,000 MiSeq reads were obtained for the isolate. The MinION data were assembled using Canu version 1.5 and polished using Nanopolish version 0.5. The MiSeq reads were mapped onto the final MinION assembly using Pilon version 1.20.

A single closed genome was constructed consisting of a chromosome of 4,344,886 bp and two plasmids of 165,144 bp and 122,411 bp, respectively. Annotation was performed using the NCBI Prokaryotic Genome Annotation Pipeline (2013), which predicted a total of 4,015 coding sequences. Interestingly, comparative analysis of the plasmid sequences with those previously identified in other *Legionella* spp., showed that the 122-kb plasmid (CP025493) contained a 40,243-bp fragment of the *L. longbeachae* NSW150 plasmid, providing further evidence of gene transfer across *Legionella* spp. through mosaic plasmids, as first suggested by Bacigalupe et al. (7).

### Accession number(s).

MinION and Illumina MiSeq sequence reads described here have been deposited at NCBI/GenBank under the BioProject number PRJNA384907. The whole-genome sequence described here has been deposited at NCBI/GenBank under the accession numbers CP025491 (chromosome), CP025492 (plasmid 1), and CP025493 (plasmid 2).
